# An Adaptive Ridge Procedure for *L*_0_ Regularization

**DOI:** 10.1371/journal.pone.0148620

**Published:** 2016-02-05

**Authors:** Florian Frommlet, Grégory Nuel

**Affiliations:** 1 Department of Medical Statistics (CEMSIIS), Medical University of Vienna, Spitalgasse 23, A-1090 Vienna, Austria; 2 National Institute for Mathematical Sciences (INSMI), CNRS, Stochastics and Biology Group (PSB), LPMA UMR CNRS 7599, Université Pierre et Marie Curie, 4 place Jussieu, 75005 Paris, France; Cleveland Clinic Lerner Research Institute, UNITED STATES

## Abstract

Penalized selection criteria like AIC or BIC are among the most popular methods for variable selection. Their theoretical properties have been studied intensively and are well understood, but making use of them in case of high-dimensional data is difficult due to the non-convex optimization problem induced by *L*_0_ penalties. In this paper we introduce an adaptive ridge procedure (AR), where iteratively weighted ridge problems are solved whose weights are updated in such a way that the procedure converges towards selection with *L*_0_ penalties. After introducing AR its specific shrinkage properties are studied in the particular case of orthogonal linear regression. Based on extensive simulations for the non-orthogonal case as well as for Poisson regression the performance of AR is studied and compared with SCAD and adaptive LASSO. Furthermore an efficient implementation of AR in the context of least-squares segmentation is presented. The paper ends with an illustrative example of applying AR to analyze GWAS data.

## Introduction

Methods for performing variable selection, particularly in a high dimensional setting, have undergone tremendous development over the last two decades. Of particular importance in this context is penalized maximum likelihood estimation, which can be divided in selection methods based on generalized information criteria and regularization methods [1]. The former use a penalty which depends on the number of estimated parameters, sometimes called *L*_0_ penalty, and include the classical information criteria AIC [2] and BIC [3]. Their asymptotic properties have been thoroughly studied and are well understood when the number of potential regressors is fixed (see for example [4] and citations given there). Specifically BIC is known to yield a consistent model selection rule, which means that as the sample size goes to infinity the probability of selecting the true model goes to 1. However, this is no longer true in a high dimensional setting, where under sparsity both AIC and BIC tend to select too large models [5]. As a consequence a number of different modifications of BIC have been suggested, for example mBIC [6, 7] which is designed to control the family wise error rate (FWER), mBIC2 [8, 9] controlling the false discovery rate, or EBIC [10] for which consistency under certain asymptotic conditions has been shown even when the number of regressors is allowed to be larger than the sample size.

Thus from a theoretical perspective it is rather appealing to perform model selection using generalized information criteria. However, the corresponding optimization problem is notoriously difficult due to the non-convexity and discontinuity of the *L*_0_ penalty. It is an NP hard problem to find the model which minimizes a specific information criterion, and in general already for a moderate number of say fifty variables it becomes computationally infeasible to guarantee finding the optimal solution. Another problem often associated with *L*_0_ penalties is the instability of selected solutions [11]. A possible workaround is to report not only one model which minimizes the criterion, but a number of good models which have been found for example with some evolutionary algorithms [12]. In any case the approach remains extremely computer intensive and time consuming for high-dimensional data sets.

Regularization methods can serve as an alternative, where penalties are not based on the number, but rather on the size of coefficients. A prominent example is bridge regression [13] which uses penalties of the form $\sum_{i}\beta_{i}^{q}$, where *β*_*i*_ are the coefficients of the model to be estimated. Special cases are ridge regression [14] for *q* = 2 and the LASSO [15] for *q* = 1, whereas for *q* → 0 the penalty of bridge regression converges towards the *L*_0_ penalty of generalized information criteria. It has been shown that only for *q* ≤ 1 bridge regression can perform variable selection [16], on the other hand only for *q* ≥ 1 its penalty is convex and therefore allows for relatively simple optimization algorithms. This partly explains the huge interest that the LASSO (*q* = 1) has received in recent years (see [17] for a comprehensive treatment).

The LASSO has very nice properties in terms of prediction, but as a model selection procedure it is consistent only under rather restrictive assumptions [17, 18]. Specifically for strongly correlated regressors it can perform quite poorly, and a number of non-convex penalties have been studied to achieve sparser solutions [19], among them the smoothly clipped absolute deviation (SCAD) [20] and the minimax concave penalty (MCP) [21]. Furthermore the coefficient estimates of the LASSO are severely biased due to shrinkage. An interesting procedure to overcome these deficits is the adaptive LASSO [22], which makes use of a weighted *L*_1_ norm penalty resulting in a similar convex optimization problem as the original LASSO. With suitable choice of the weights the adaptive LASSO was shown to have the oracle property, which means that it is both consistent and the nonzero coefficients are estimated as well as when the correct model was known. The weights for the adaptive LASSO can be obtained with some initial LASSO estimates, and if this procedure is further iterated one obtains a multi-step adaptive LASSO [23, 24]

Already much earlier Grandvalet showed that the LASSO estimate can be obtained via some weighted ridge regression [25, 26]. He called his procedure adaptive ridge regression, of which a slightly modified version has been recently applied to detect rare variants in genome wide association studies [27]. In this article we want to study a different adaptive ridge procedure, which was recently proposed [28–30] with the aim of approximating *L*_0_ penalties. This Adaptive Ridge (AR) procedure is somewhat similar to the multi-step adaptive LASSO, in the sense that the weights are iteratively adapted; but in each iteration weighted ridge regression is performed instead of weighted LASSO, which is computationally much easier.

The iteratively adapted weights of AR are designed in such a way that the resulting penalty converges towards the *L*_0_ penalty. Therefore the procedure is somewhat related to the seamless *L*_0_-penalty [31] and the combination of *L*_0_ and *L*_1_ penalties suggested in [32], which both represent regularized versions of the *L*_0_ penalty. However, the latter procedures rely upon non-convex optimization, which gets computationally rather difficult for large-scale problems as well as for applications beyond linear regression. In contrast each iteration of the suggested AR is extremely fast, and we will see that the method also performs really well in some non-linear examples.

The main purpose of this article is to look more systematically into the statistical properties of the AR procedure proposed in [29]. After introducing the general procedure we will first focus on the special case of linear regression. In particular we will provide some theoretical results under an orthogonal design which elucidates the amount of shrinkage introduced by AR. In extensive simulation studies we will then show to which extent these results also apply for linear regression with more general design matrices, as well as for some generalized linear models. We compare the performance of AR with SCAD and multi-step adaptive LASSO both in terms of runtime and correct classification of regressors. Furthermore we will introduce a new implementation of AR for least squares segmentation which is much more efficient than the one used originally by Rippe et al. in [29]. Finally we illustrate the usefulness of AR in the context of real GWAS data.

## The Adaptive Ridge procedure

### The Problem

Consider a parametric model with parameter vector $\beta \in R^{d}$, in combination with a $C^{2}$ convex contrast $C:R^{d}\to R$. The most common examples of contrasts *C*(***β***) are the residual sum of squares, or minus twice the log-likelihood of a given model, but more general functions like pseudo-likelihood related quantities are conceivable. For all 0 ≤ *q* ≤ 2, *λ* ≥ 0 we introduce the penalized contrast

$$\begin{array}{c} C_{\lambda ,q}\left(\beta \right)\overset{\Delta }{=}C\left(\beta \right)+\lambda {∥\beta ∥}_{L_{q}}^{q}\;. \end{array}$$ (1)

**Remark 1**. *One can easily replace* ***β*** *in the penalty term by any linear transformation* ***Dβ*** *allowing to consider wider generalizations of penalty forms. For example one might consider a subspace extraction such that* $D\beta =\beta_{J}$ *for a given set* J⊂{1,2,…,d}, *or a difference matrix such that* ***Dβ*** = (*β*_1_ − *β*_2_, *β*_2_ − *β*_3_, …, *β*_*d*−1_ − *β*_*d*_)^*T*^ (*where* ^*T*^ *denotes the transpose operator). We will make use of this only when discussing least squares segmentation. All the results obtained previously can be straightforwardly extended for penalties of the form* ${∥D\beta ∥}_{Lq}^{q}$, *but the generalization is omitted for the sake of simplicity*.

The objective of this paper is to minimize the penalized contrast of Eq (1) in order to obtain:

$$\begin{array}{c} \hat{\beta }\overset{\Delta }{=}\arg\min_{\beta }C_{\lambda ,q}\left(\beta \right). \end{array}$$ (2)

This relates to Bridge regression for *q* > 0 [13], with the special cases of ridge regression for *q* = 2, and LASSO for *q* = 1 [15]. Note that if *q* > 1, the penalized contrast is both convex and $C^{2}$ and the problem can be easily solved with straightforward convex optimization (Gradient descent, Newton-Raphson, etc.). For *q* = 1, the problem is still convex but with derivative singularities that makes the optimization problem more delicate but still tractable (coordinate descent [33], gradient LASSO [34], etc.). If 0 ≤ *q* < 1, the penalized contrast is not convex anymore and the problem is much more challenging [19]. For the limiting case *q* = 0 one obtains for suitable choices of *λ* the classical model selection criteria AIC and BIC. Only for very small *p* it is possible to apply exact algorithms which guarantee to find the minimal solution [35], whereas for *p* > 20 one essentially has to use heuristic search strategies like stepwise selection procedures. However, variable selection based on *L*_0_ penalties is believed to be optimal for achieving sparsity and unbiasedness, and therefore there is much interest to find efficient algorithms which minimize *C*_*λ*,*q*_ also in case of *q* = 0.

### The Suggested Solution

Recently Rippe et al. [29] suggested a method for visualizing changes of copy number variation along the chromosome which is based on an iterative procedure to minimize residual sum of squares with *L*_0_ penalties. We will adapt this procedure to our setting of penalized likelihoods and discuss it in a slightly more general form. The idea is to obtain $\hat{\beta }$ through an iterative weighted fixed-point procedure. For any *λ* ≥ 0 and any non-negative weight vector $w\in R_{+}^{p}$ we introduce the function:

$$\begin{array}{c} F_{\lambda ,w}\left(\beta \right)\overset{\Delta }{=}C\left(\beta \right)+\frac{\lambda }{2}\beta^{T}\text{diag}\left(w\right)\beta =C\left(\beta \right)+\frac{\lambda }{2}\sum_{j=1}^{d}w_{j}\beta_{j}^{2}\;, \end{array}$$ (3)

where diag(***w***) is the diagonal matrix with weights ***w*** on its diagonal. We are now ready to introduce our Adaptive Ridge procedure:

**Definition 1 (AR)**. *For any* *λ* > 0 *and* 0 ≤ *q* < 2, *the* *L*_*q*_ *Adaptive Ridge sequences* ***β***^(*k*)^ *and* ***w***^(*k*)^ *are defined by the initialization* ***w***^(0)^ = **1**, *and for* k∈N *by*:

$$\begin{array}{c} \beta^{\left(k\right)}=\arg\min_{\beta }F_{\lambda ,w^{\left(k-1\right)}}\left(\beta \right) \end{array}$$ (4)

$$\begin{array}{c} w^{\left(k\right)}={\left({\left|\beta^{\left(k\right)}\right|}^{\gamma }+\delta^{\gamma }\right)}^{(q-2)/\gamma } \end{array}$$ (5)

*where* Eq (5) *is defined component-wise, and depends on the constants* *δ* > 0 *and* *γ* > 0.

Eq (4) is just a weighted version of ridge regression, which is usually fast to solve. Note that for *q* = 2 one always has ***w***^(*k*)^ = **1** and thus the procedure is not really iterative. In contrast for *q* < 2, ***w***^(*k*)^ *does* depend on the iteration step *k*, and in case of convergence of the sequence ***β***^(*k*)^ we will write $\beta^{\left(k\right)}\to \tilde{\beta }$.

The form of the weights ***w***^(*k*)^ of Eq (5) is motivated by the heuristic consideration that at least formally the penalty term of Eq (3) converges towards the penalty term of Eq (1),

$$\begin{array}{c} \beta^{(k)T}\text{diag}(w^{\left(k-1\right)})\beta^{\left(k\right)}\underset{k\to \infty }{\to }\sum_{j=1}^{d}\frac{{\tilde{\beta }}_{j}^{2}}{{\left({|{\tilde{\beta }}_{j}|}^{\gamma }+\delta^{\gamma }\right)}^{\frac{\left(2-q\right)}{\gamma }}}\approx \sum_{j=1}^{d}{|{\tilde{\beta }}_{j}|}^{q}={∥\tilde{\beta }∥}_{L_{q}}^{q}\;. \end{array}$$ (6)

For *q* = 1 one obtains in the limit the LASSO penalty by iteratively solving weighted ridge problems, which has been exactly the motivation of the Adaptive Ridge approach introduced in [25]. However, the main aim of our Adaptive Ridge procedure AR is not to approximate the LASSO, but to focus on 0 ≤ *q* < 1, and especially on the case *q* = 0. As a consequence our AR is more similar in spirit to the multi-step adaptive LASSO discussed in [23] and [24], where iteratively the weights of the ℓ_1_ penalty are updated using formulas which are very similar to Eq (5). More precisely both references make use of *γ* = 1, whereas we will later recommend to work with *γ* = 2. Furthermore one finds *δ* = 0 in [23], whereas [24] introduces *δ* > 0 for numerical stability. Again we will discuss the exact choice of *δ* in our procedure below.

The main advantage of our AR approach compared with the multi-step adaptive LASSO is that solving a ridge problem in each iteration is much easier than solving a LASSO problem. While AR works for any *q* < 1 we will focus here on the case *q* = 0, which corresponds to a number of widely used variable selection criteria, and for which minimizing Eq (1) is particularly difficult. In fact this optimization problem is NP hard with growing *p*, and thus it is very useful to have a good approximate procedure.

### Numerical considerations

The Definition (5) of the weights *w*_*j*_ is very intuitive but from an algorithmic perspective its algebraic form is not ideal. Typically the terms *β*^(*k*)^ and *δ* will be of different order and computing the sum of |*β*^(*k*)^|^*γ*^ and *δ*^*γ*^ in floating point arithmetics becomes problematic. The following formula to update the weights is algebraically equivalent to Eq (5) but avoids any numerical instabilities:

$$\begin{array}{c} w_{j}=\{\begin{array}{cc} \delta^{q-2}\exp[\frac{q-2}{\gamma }\text{log1p}({\left|\frac{\beta_{j}}{\delta }\right|}^{\gamma })] & \text{if}\;|{\tilde{\beta }}_{j}|\leq \delta \\ {|{\tilde{\beta }}_{j}|}^{q-2}\exp[\frac{q-2}{\gamma }\text{log1p}({\left|\frac{\delta }{\beta_{j}}\right|}^{\gamma })] & \text{if}\;|{\tilde{\beta }}_{j}|>\delta \end{array} \end{array}$$ (7)

where log1*p* is the classical function defined by $\text{log1p}\left(u\right)\overset{\Delta }{=}\log\left(1+u\right)$ (for all *u* > −1) for which stable implementations are publically available.

According to Definition 1 the AR procedure depends on two parameters, *δ* and *γ*. The choice of *δ* calibrates which effect sizes are considered as relevant. If *β*_*j*_ < *δ* the corresponding weight *w*_*j*_ will become large. Eventually one will obtain in the limit ${\tilde{\beta }}_{j}\approx 0$, and thus also $w_{j}{\tilde{\beta }}_{j}^{2}\approx 0$. On the other hand for *β*_*j*_ ≫ *δ* it holds that $w_{j}{\tilde{\beta }}_{j}^{2}\approx {|{\tilde{\beta }}_{j}|}^{q}$. A choice of *δ* = 0 (like in [23]) might then appear to be reasonable, but our numerical experiments show that it leads to numerical instabilities and that a small *δ* > 0 (like in [24, 29]) performs noticeably better. Simulation results (not presented in this manuscript) suggest that in case of standardized data the procedure is not particularly sensitive to the exact choice of *δ*, which coincides with the findings of [24] in case of adaptive LASSO. Throughout this paper we will thus work with *δ* = 10^−5^.

**Fig 1:**
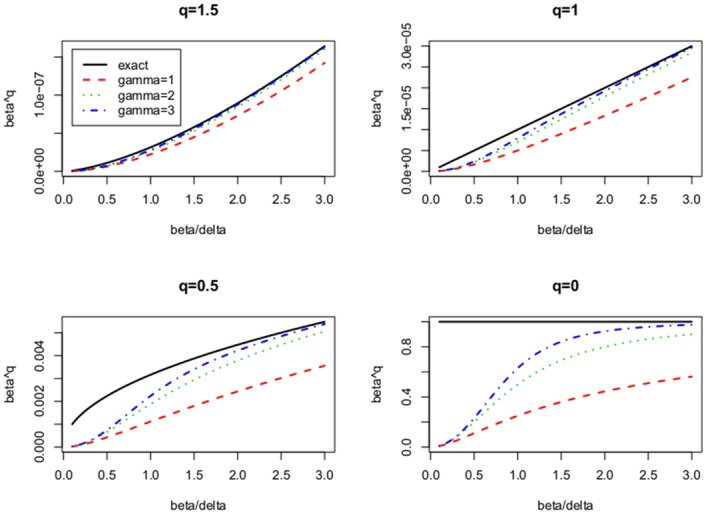
Approximation of |*β*_*j*_|^*q*^ by the function $\beta_{j}^{2}{\left({|\beta_{j}|}^{\gamma }+\delta^{\gamma }\right)}^{(q-2)/\gamma }$ in dependence of the parameter *γ* ∈ {1, 2, 3}. The x-axis is at the scale of *δ*. The four panels illustrate the cases *q* = 1.5, 1, 0.5, 0.

The second parameter *γ* determines the quality of the approximation $w_{j}{\tilde{\beta }}_{j}^{2}\approx {|{\tilde{\beta }}_{j}|}^{q}$. Fig 1 illustrates for several choices of *q* the shape of $w_{j}{\tilde{\beta }}_{j}^{2}$ depending on the parameter *γ*. Clearly for increasing values of *γ* the approximation is getting closer to the desired thresholding step function. In simulations not presented here we observed dramatic improvement of the performance of AR by raising the parameter from *γ* = 1.0 (like in [23–25]) to *γ* = 2.0 (like in [29]), while further increasing of *γ* did not yield much more benefit.

For the rest of the paper we will focus on the variable selection case *q* = 0, and stick with the choice *δ* = 10^−5^ and *γ* = 2. The Adaptive Ridge Regression procedure for *L*_0_ regularization is therefore defined by the following (component-wise defined) weighting scheme:

$$\begin{array}{c} w^{\left(k\right)}={\left({\left(\beta^{\left(k\right)}\right)}^{2}+\delta^{2}\right)}^{-1}. \end{array}$$ (8)

### Shrinkage for Linear Regression

Our first objective is to study AR theoretically in the context of linear regression. Thus consider the model

$$\begin{array}{c} y=X\beta +\varepsilon \;, \end{array}$$ (9)

where $y\in R^{n}$, $X=\left(X_{1},\cdots ,X_{p}\right)\in R^{n\times p}$ and $\beta \in R^{p}$. The error terms are assumed to be i.i.d. normal, $\varepsilon_{i}∼N\left(0,\sigma^{2}\right)$. Furthermore let ***y*** be centralized, that is $\sum_{i=1}^{n}y_{i}=0$, and let all regressors be centralized and standardized such that $X_{j}^{T}X_{j}=n$. Specifically this means that we consider only models without intercept.

Clearly the log-likelihood of Model (9) is of the form

$$\begin{array}{c} \ell \left(\beta ,\sigma^{2}\right)=\text{const.}-n\log\sigma -\frac{1}{2\sigma^{2}}{\left(X\beta -y\right)}^{T}\left(X\beta -y\right)\;. \end{array}$$

Then −2ℓ takes the role of the convex contrast *C* in Eq (1), and in case of known error variance *σ*^2^ we obtain (after neglecting constants)

$$\begin{array}{c} C\left(\beta \right)=\frac{1}{\sigma^{2}}{\left(X\beta -y\right)}^{T}\left(X\beta -y\right)\overset{\Delta }{=}\frac{\text{RSS}(\beta )}{\sigma^{2}}\;. \end{array}$$

Variable selection with classical model selection criteria like AIC or BIC becomes a special case of Eq (1) with *q* = 0. More specifically let a model be defined by the set of non-zero coefficients *M* = {*j*: *β*_*j*_ ≠ 0}. Then Eq (1) becomes

$$\begin{array}{c} C_{\lambda ,0}\left(\beta \right)=\frac{1}{\sigma^{2}}\text{RSS}\left(\beta \right)+\lambda \left|M\right|\;, \end{array}$$ (10)

which for a given model *M* is clearly minimized at ${\hat{\beta }}_{M}$, the maximum likelihood estimate with respect to the given model.

We now want to compare variable selection based on Eq (10) with the AR procedure defined by Eqs (4) and (8). It is straight forward to see that for linear regression Eq (4) can be written as an explicit dynamic system,

$$\begin{array}{c} {\tilde{\beta }}^{\left(k\right)}={\left(X^{T}X+\tilde{\lambda }\sigma^{2}\;\text{diag}\left(w\right)\right)}^{-1}X^{T}y\;. \end{array}$$ (11)

Our major theoretical result is concerned with shrinkage of coefficients resulting from the AR procedure. It turns out that the non-zero coefficients of $\tilde{\beta }=\lim{\tilde{\beta }}^{\left(k\right)}$ obtained via Eq (11) are smaller in absolute terms than the maximum likelihood estimates ${\hat{\beta }}^{M}$ of a model *M* containing exactly the same non-zero coefficients as $\tilde{\beta }$. Closely related is the fact that AR with parameter $\tilde{\lambda }=\lambda$ does not directly correspond to variable selection based on minimizing *C*_*λ*,0_(***β***), but that a smaller value of $\tilde{\lambda }$ must be chosen. Theorem 1 gives the precise relationship between AR and exact model selection based on information criteria for the case of orthogonal regressors. Subsequently in the Results Section we will illustrate the behavior of AR for a variety of more complex models based on comprehensive simulation studies.

The case of orthogonal regressors is of limited use for most applications, but it allows a detailed analysis of the shrinkage properties of the AR procedure. Specifically the following relationship between the penalties *λ* and $\tilde{\lambda }$ must hold to guarantee that AR with penalty $\tilde{\lambda }$ gives the same results as selection based on the penalized likelihood with penalty *λ*.

**Theorem 1**. *In the context of linear regression under orthogonality performing AR with* $\tilde{\lambda }$ *corresponds to minimizing* Eq (10) *with* $\lambda =4\tilde{\lambda }$.

The proof is given in full detail in S1 Proof.

**Remark**: *The result holds under the condition that* $\tilde{\lambda }<n/\sigma^{2}$. *In practice this seems to be no huge restriction. To give some examples, the penalties of AIC, BIC and mBIC are* *λ* = 2, *λ* = log *n* *and* *λ* = log(*np*^2^/4^2^), *respectively. As long as* *y* *is reasonably scaled the condition* *λ*/4 < *n*/*σ*^2^ *will always apply.*

## Results

In this section we will provide results from simulation studies and from a real data analysis to illustrate the performance of AR in the context of different models. The simulation scenarios include linear and generalized linear models as well as the problem of least squares segmentation. The real data sets stem from a large genome-wide association study [36] concerned with metabolic traits in a Finnish cohort.

### Linear Regression

The initial simulation scenarios will be concerned with different linear regression models, where we are interested to which extent the results of Theorem 1 still hold for more general correlation structures between regressors. For the non-orthogonal case a full analysis of the dynamical system Eq (11) becomes way more complicated, because it cannot be reduced any longer to independent analysis for the individual coefficients. Instead of attempting to obtain analytic results we will focus here on illustrating the most important features of AR by presenting results from simulations. Before that we only want to mention that as a simple consequence of Eq (11) it always holds that

$$\begin{array}{c} ∥{\tilde{\beta }}^{\left(k\right)}∥\leq ∥{\left(X^{T}X\right)}^{-1}X^{T}y∥, \end{array}$$ (12)

and thus the sequence of ${\tilde{\beta }}^{\left(k\right)}$ remains bounded. However, it turns out that the mapping underlying the dynamic system ${\tilde{\beta }}^{\left(k\right)}$ is usually not a contraction, and therefore theoretical convergence results are rather hard to obtain. In fact changing the initial value of the weights ***w***^(0)^ can have some effect on the limit of ${\tilde{\beta }}^{\left(k\right)}$, though usually the obtained solutions are not too different from each other.

**Fig 2:**
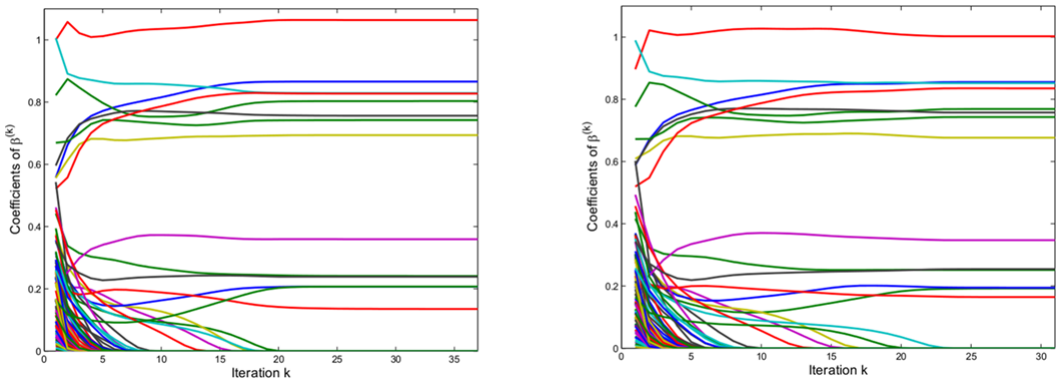
Convergence of procedure for one simulated instance where the standard initial value (left panel) is compared with a different choice of ***w***^(0)^ (right panel).

Fig 2 provides a typical example that illustrates the behavior of AR for the general linear case. We simulated one instance according to Eq (9) with *p* = *n* = 100, where the correct model had k* = 24 regressors. The first plot uses our standard initial value $w_{j}^{\left(0\right)}=1$ for all components, whereas in the second plot the components of the initial value are randomly chosen between 1/2 and 3/2. The models resulting from the two starting points differ only in one regressor, where a true positive detected by the second model is substituted in the first model by a false positive. Otherwise both models contain the same non-zero coefficients, for which estimates can also slightly differ. For this instance trying further random initial values of $w_{j}^{\left(0\right)}∼U\left(0.5,1.5\right)$ provided a third limiting model which added one false positive to the second model. Interestingly the model obtained with the second starting point which was doing best in terms of misclassification had the largest BIC criterion (141.03), while the other two models had almost identical BIC criterion (140.06 and 140.07). In general our experience with simulations shows that although the limit of the AR procedure depends on the starting point, the different solutions obtained will have very similar values of the selection criterion that one attempts to approximate. In fact the instability of solutions does not come as a surprise bearing in mind that variable selection based on information criteria is well known to suffer from instabilities with respect to small changes within the data [1].

Note that for the two examples shown in Fig 2 any component of the sequence ${\tilde{\beta }}^{\left(k\right)}$ which once has approached zero also remains there. This is actually always the case because for small ${\tilde{\beta }}_{j}^{\left(k\right)}$ the corresponding weight *w*_*j*_ becomes very large, and the matrix $X^{T}X+\tilde{\lambda }\sigma^{2}\text{diag}\left(w\right)$ becomes essentially orthogonal with respect to the *j*-th component. The majority of coordinates converging to zero does so within less than 10 iterations, but there are some exceptions for which convergence to zero takes substantially longer. The instability of the AR model as a function of the initial values appears to depend mainly on the behavior within the first few iterations, where for the majority of coefficients it becomes clear whether they are selected or not. In practical applications it is sometimes desired to perform model selection only on a certain subset of regressors. This can be rather easily achieved within the AR procedure: One can force some *β*_*j*_ to stay in the model by setting its corresponding initial weight *w*_*j*_ to 0 and keeping it then fixed at 0 for all iterations.

We will next consider three different simulation scenarios, where the first two are aiming at verifying to which extent the result of Theorem 1 can be extended to non-orthogonal regressors. In the third scenario we will then compare the performance of AR with other model selection approaches.

#### Simulation Scenario: Correlated regressors

In our first set of simulations we consider with *p* = 15 a relatively small number of regressors. This allows for a systematic examination of the performance of AR compared with all subset selection, which is for *p* = 15 still conveniently possible. Thus we can directly evaluate to which extent the relationship $\lambda =4\tilde{\lambda }$ of Theorem 1 holds for the non-orthogonal case. We consider correlation structures from compound symmetry and auto regressive models letting a parameter *ρ* vary between 0 and 0.8, where *ρ* specifies pairwise correlation between neighboring covariates for auto regression (Scenario 2), and pairwise correlation between all regressors for compound symmetry (Scenario 1). For each scenario we simulate 500 traits for *n* = 50 individuals based on linear models with 5 regressors having nonzero coefficients. The effects are all chosen to be *β*_*j*_ = 0.5, which equals half of the predefined standard deviation *σ* = 1. Regressors entering the model were chosen to be *j* ∈ {1, …, 5} for Scenario 1, and *j* ∈ {2, 5, 8, 11, 14} for the second scenario. Selection based on BIC is compared with AR using parameter *λ* = log(*n*)/4, that is we use the relationship $\lambda =4\tilde{\lambda }$ as suggested by Theorem 1.

**Fig 3:**
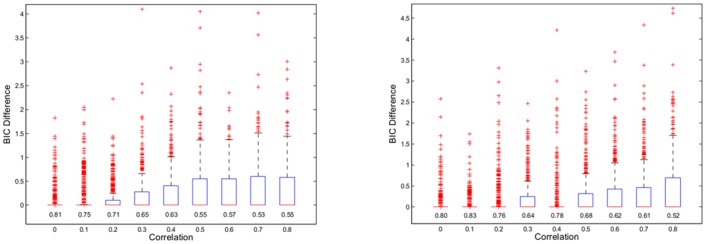
Difference of BIC between model obtained with AR and best model. The numbers below the boxplots give the relative frequency of simulation runs in which AR gave the optimal solution. In the left panel we used a correlation structure of compound symmetry, and we used an auto regression model in the right panel.

Fig 3 illustrates to which extent AR yields the optimal model according to BIC. For small correlations AR gives in the majority of cases the same model as all subset selection, which starts to change only for *ρ* ≥ 0.3. In Scenario 2 AR yields more often the optimal model than in Scenario 1, when comparing results at the same level of pairwise correlation. Clearly a compound symmetry model provides in general more correlation between regressors than an autoregressive model, and we might conclude that AR differs increasingly from all subset selection the farther away one gets from orthogonality.

Interestingly from a statistical point of view AR seems to perform almost better than all subset selection based on BIC. For the majority of cases AR has less misclassifications than all subset selection (see Table 1). Specifically for Scenario 1 AR tends to have larger power to detect the correct regressors, while controlling the Type I error at a similar rate like BIC. On the other hand in Scenario 2 AR tends to give less Type I errors, while having similar power to BIC. In summary one might conclude that for *p* < *n* (at least in these two scenarios) the choice of $\lambda =4\tilde{\lambda }$ from Theorem 1 worked quite well even in the non-orthogonal case.

**Table 1:** First Simulation on linear regression. Comparison of the performance of all subset selection (BIC) with AR in terms of power, number of false positives (FP), false discovery rate (FDR) and number of misclassifications (Mis). For Scenario 1 the correlation (Corr) refers to pairwise correlation between all regressors, for Scenario 2 only for neighboring regressors.

	Power		FP		FDR		Mis	
Corr	BIC	AR	BIC	AR	BIC	AR	BIC	AR
**Scenario 1**:								
0.0	0.85	0.83	0.54	0.52	0.11	0.10	1.30	1.39
0.1	0.81	0.84	0.61	0.62	0.11	0.11	1.54	1.42
0.2	0.82	0.86	0.56	0.51	0.10	0.09	1.47	1.23
0.3	0.79	0.83	0.65	0.66	0.13	0.12	1.71	1.50
0.4	0.75	0.80	0.61	0.62	0.12	0.12	1.86	1.60
0.5	0.68	0.74	0.79	0.73	0.17	0.15	2.38	2.02
0.6	0.66	0.72	0.61	0.65	0.14	0.14	2.30	2.04
0.7	0.56	0.62	0.71	0.76	0.19	0.18	2.90	2.68
0.8	0.49	0.54	0.84	0.87	0.25	0.24	3.41	3.19
**Scenario 2**:								
0.0	0.85	0.83	0.54	0.47	0.10	0.10	1.29	1.35
0.1	0.86	0.88	0.55	0.53	0.10	0.09	1.24	1.11
0.2	0.81	0.80	0.62	0.54	0.13	0.11	1.58	1.52
0.3	0.67	0.62	0.70	0.65	0.19	0.18	2.37	2.56
0.4	0.88	0.88	0.74	0.72	0.13	0.13	1.36	1.32
0.5	0.81	0.83	0.84	0.79	0.17	0.15	1.81	1.62
0.6	0.80	0.80	0.91	0.93	0.18	0.18	1.93	1.94
0.7	0.72	0.75	1.00	0.89	0.21	0.18	2.41	2.13
0.8	0.61	0.65	1.30	1.24	0.30	0.28	3.27	3.00

#### Simulation Scenario: High-dimensional setting

A large number of recent statistical applications are confronted with the challenging task of model selection when *p* > *n*. Here we perform simulations under the assumption that regressors are independent normally distributed variables. Sample size was fixed with *n* = 100, while for the growing number of potential regressors we considered *p* ∈ {100, 250, 500, 1000, 2500, 5000, 10000}. For each setting 1000 models of size k* = 24 were simulated from Eq (9), with normally distributed random effect sizes *β*_*j*_ ∼ *N*(0, 0.5), *j* ∈ {1, …, 24}, and again an error standard deviation of *σ* = 1. Keeping k* fixed gives with growing *p* an increasingly sparse situation. Hence the model selection criterion mBIC is more appropriate than BIC (see [7]), but here we are mainly interested in studying the properties of AR and will therefore show results for both criteria.

We want to compare the performance of variable selection using simple stepwise search strategies for the two information criteria BIC and mBIC with their respective AR procedures. Our stepwise procedure is fairly simple. It starts with a model including the best 40 regressors according to marginal test statistics. Then greedy backward elimination is performed all the way down to a model of size one. That model along the way which minimizes the criterion in question is then considered as the starting point for some final greedy forward selection which is performed till no more improvement of the criterion is obtained. For the AR procedure we use again the relationship $\lambda =4\tilde{\lambda }$ from Theorem 1. Before applying AR the top 100 regressors were preselected based on marginal tests, which noticeably improved the performance of AR.

**Fig 4:**
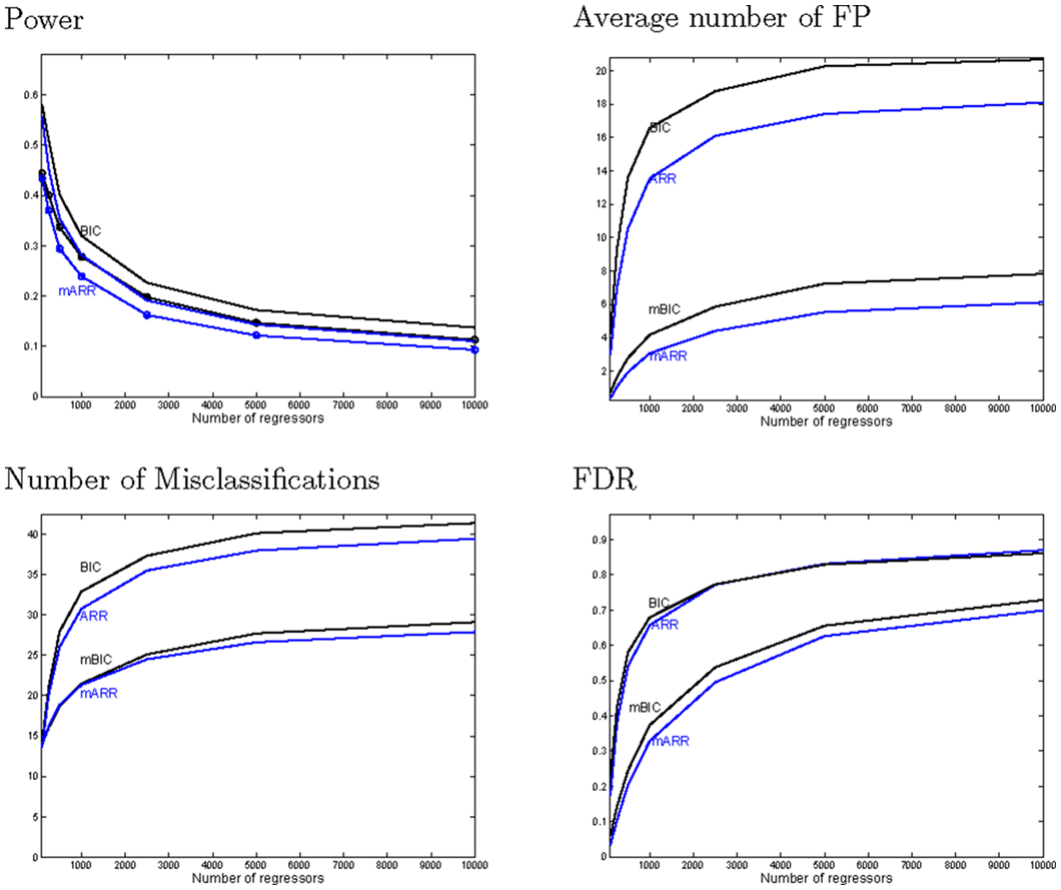
Comparison of model selection performance based on some stepwise selection procedure for BIC and mBIC with the corresponding AR procedures. The four panels show the average over 1000 simulation runs of power, number of false positives, number of misclassifications and false discovery rate as a function of the total number of potential regressors *p*. Data were simulated under a model with *k* = 24 regressors.

We start with discussing Fig 4, which compares classification characteristics of the four procedures. Only for *n* = *p* = 100 BIC and mBIC are comparable in terms of misclassification. With growing *p* BIC produces exceedingly more false positives than mBIC, which cannot be compensated by the relatively mild gain in power. Both for BIC and mBIC the AR procedure is more conservative than the corresponding stepwise selection procedure, which means that it is less powerful, but produces also less false positives. Interestingly for both criteria AR produces less misclassifications than stepwise selection.

**Fig 5:**
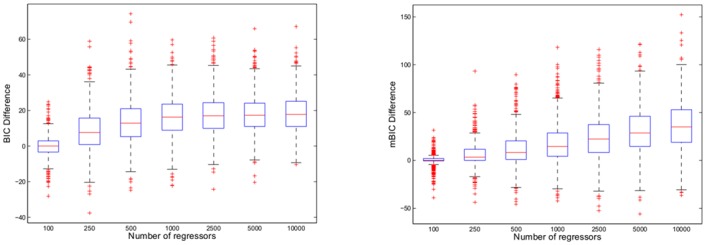
Boxplots of differences between values of selection criteria for models obtained with stepwise search strategy and with AR. The first panel shows results for BIC, the second panel for mBIC. Results are based on the same data as Fig 4.

Looking again at the differences of criteria for models obtained with stepwise selection and with AR, one can see in Fig 5 that for *p* getting larger AR tends to give models with larger values of the criterion than stepwise selection. However, even for the largest *p* there are at least some instances where AR gives better models according to each criterion than stepwise selection. For *p* = *n* = 100 AR and stepwise selection perform more or less identical, where the median of differences is almost exactly at 0. In case of BIC the median of differences increases with *p* till *p* = 1000 and then remains constant, whereas for mBIC the median of differences continues to grow also for larger values of *p*. It is interesting to observe that although for *p* > *n* AR does usually not manage to find those models that minimize the information criterion, it outperforms the corresponding stepwise selection procedure with respect to misclassification.

The fact that the AR procedure is for *p* > *n* more conservative than stepwise selection gives rise to the question whether the relationship $\lambda =4\tilde{\lambda }$ from Theorem 1 is still correct, or whether one would rather have to use in that situation more relaxed penalties to compensate for shrinkage. Our simulation results did not provide a definite answer to this question, but we will see next that it is easy to obtain solutions of AR for a whole range of $\tilde{\lambda }$ values, among which one can then choose the model which minimizes the original *L*_0_ penalty with parameter *λ*.

#### Comparison of AR with SCAD and adaptive LASSO

In the third set of simulations for linear regression we want to compare the performance of AR not only with stepwise regression, but also with two other state of the art selection methods, namely SCAD which is performed by using the ncvreg R-package [37], and adaptive LASSO by using the lqa R-package [38]. In case of adaptive LASSO we would ideally like to compare AR with a multi step version which is iterated till convergence is achieved, but up to our knowledge there is no software available which performs this task. As a compromise we perform five steps of adaptive LASSO using the lqa package and call this procedure ALASSO.

**Fig 6:**
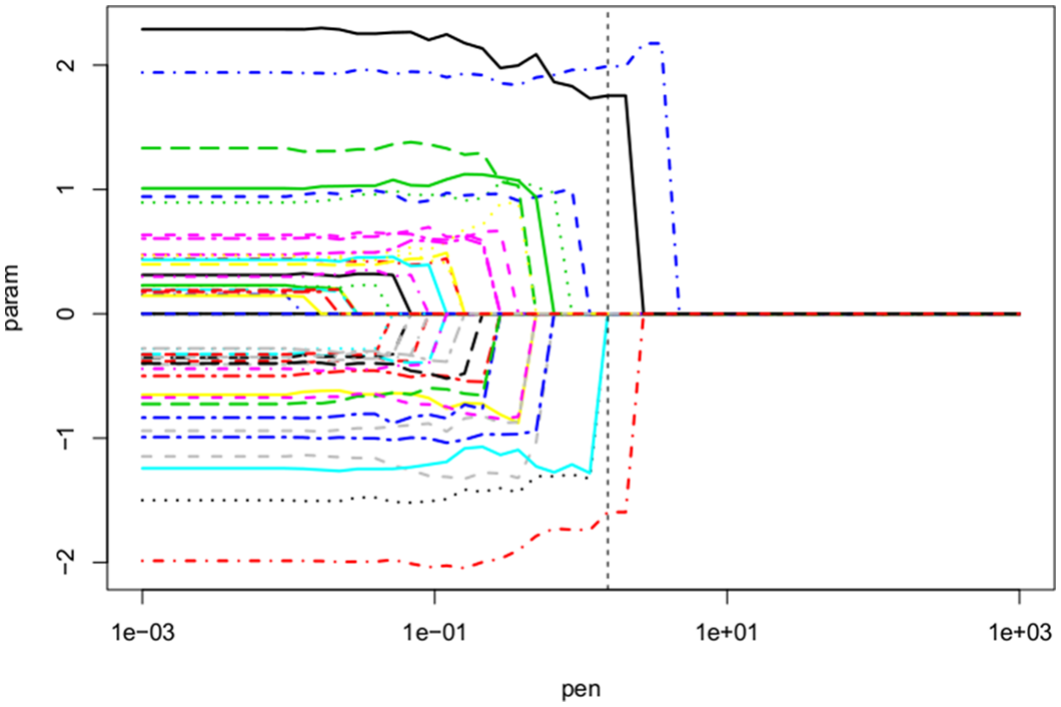
Example of a full regularization path for *L*_0_ adaptive ridge linear regression with *n* = 300, *p* = 50, and β* = 0 except for the first *k* = 10 coordinates. The covariates *X*_*i*,*j*_ and non-zero coefficients *β*_*j*_ are independently drawn from normal random variables according to $X_{i,j}∼N\left(0,0.1^{2}\right)$ and $\beta_{j}∼N\left(0,1.5^{2}\right)$.

Like in case of LASSO it is also possible for AR to take advantage of a warm start of the algorithm to obtain the full regularization path of the problem (see Fig 6). For that purpose, we start with a near null penalty *λ*, and then increase the value of the penalty using for each new penalty the previously computed weight vector *w* and parameter *β* as starting points. Obtaining the full regularization path will be of particular importance in the next section on GLM where we do not have any theoretical results like Theorem 1 telling us which $\tilde{\lambda }$ of AR corresponds to the *λ* of a given selection criterion. However, even for linear regression it is not guaranteed for the non-orthogonal case that a scaling factor 4 (like in Theorem 1) will always gives the best model. For AR, SCAD and ALASSO we will therefore compute the selection criterion for all models along the regularization path and then choose that model along the path which minimizes the criterion.

We consider two simulation scenarios, the first one with *p* = 50, the second one with *p* = 500 potential regressors. In both scenarios we have a sample size of *n* = 300. Data were simulated from standard linear regression models of size *k* = 10 and *k* = 25, respectively. The covariates *X*_*i*,*j*_ and the non-zero coefficients *β*_*j*_ were independently drawn from normal random variables according to $X_{i,j}∼N\left(0,0.1^{2}\right)$ and $\beta_{j}∼N\left(0,1.5^{2}\right)$. For the first scenario model selection was performed using BIC, whereas for the second high-dimensional scenario we applied both BIC and mBIC. For the second scenario we also performed a pre-selection step and considered only the 75 regressors with smallest p-values from marginal tests before applying any of the four selection procedures.

**Fig 7:**
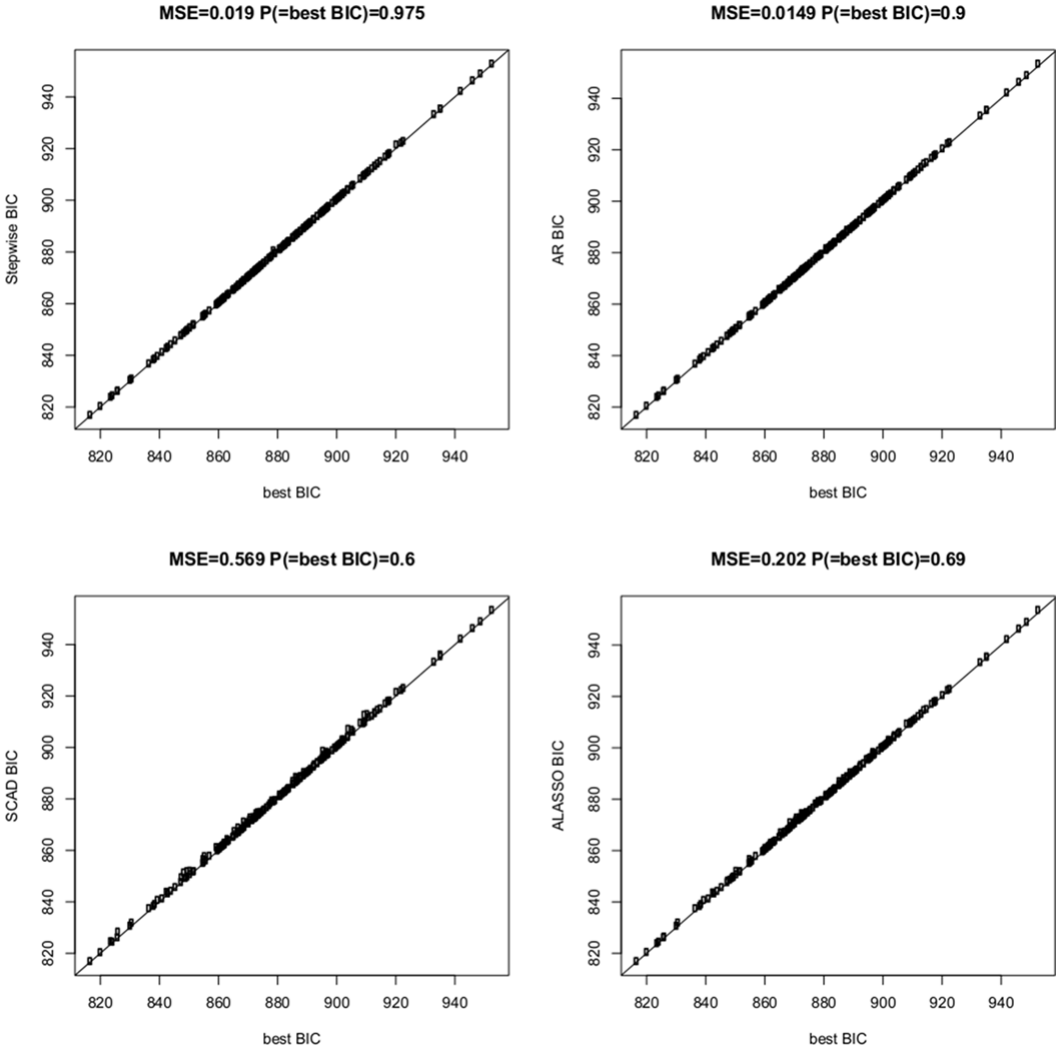
Comparison of BIC criteria of four different methods for the linear regression scenario with *n* = 300, *p* = 50, and *k* = 10 where *n* is the sample size, *p* the total number of regressors and *k* the size of the data generating model. The four panels show results for stepwise search, AR procedure, SCAD and multi-step adaptive LASSO, respectively, where criteria are compared with the best BIC criterion obtained by any of the four methods.

**Fig 8:**
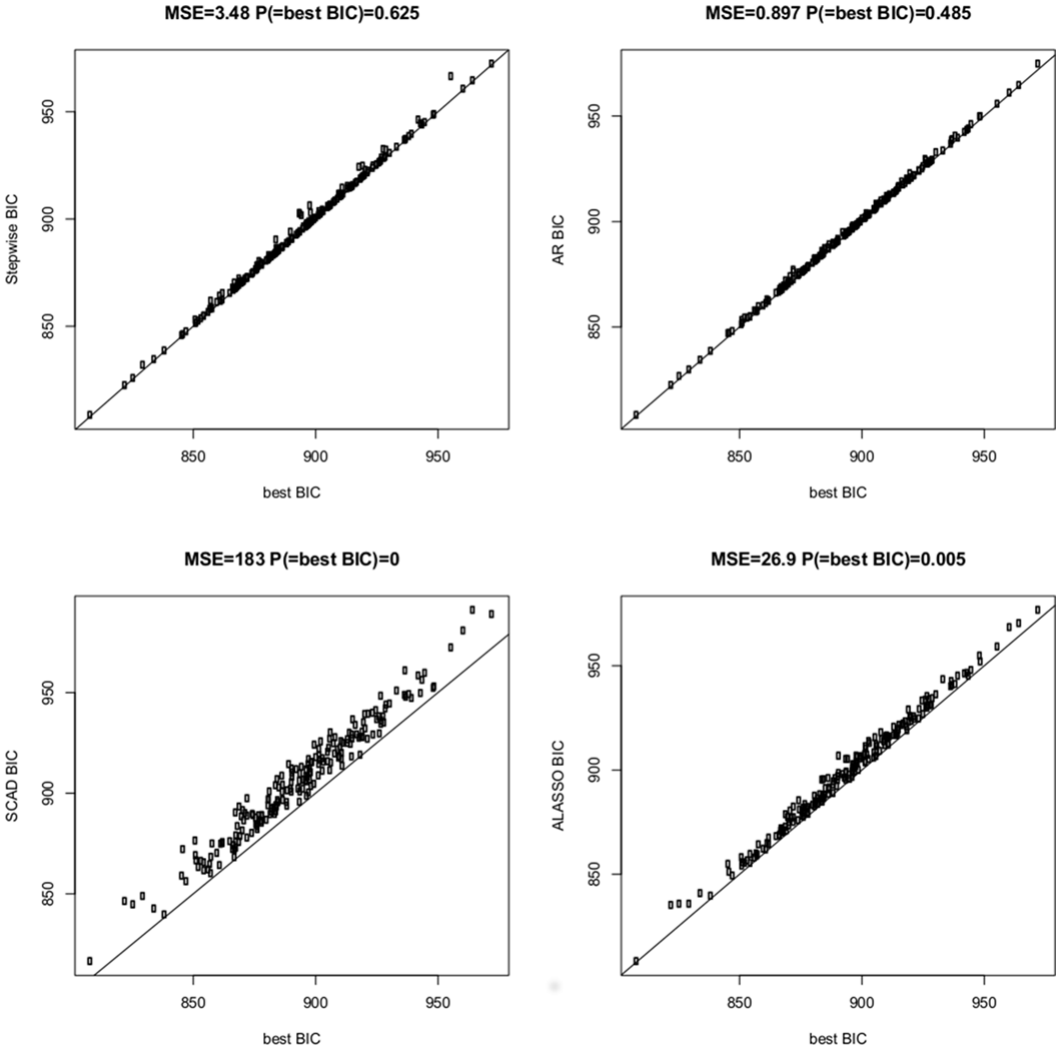
Comparison of BIC criteria of four different methods for the linear regression scenario with *n* = 300, *p* = 500, and *k* = 25 where *n* is the sample size, *p* the total number of regressors and *k* the size of the data generating model. The four panels show results for stepwise search, AR procedure, SCAD and multi-step adaptive LASSO, respectively, where criteria are compared with the best BIC criterion obtained by any of the four methods.

Table 2 as well as Figs 7 and 8 summarize the results from this simulation study. First note that AR was always only a little bit slower than SCAD, but much faster than stepwise selection or ALASSO. In the majority of cases stepwise selection gave the model with the lowest criterion, though there were a number of instances where AR gave models with smaller criterion than SW, specifically in the high-dimensional scenario when selecting with BIC. For the first scenario with *p* = 50 differences of BIC obtained with the four methods were relatively small (see Fig 7), whereas for *p* = 500 ALASSO and especially SCAD tended to yield models with substantially larger BIC value than AR and stepwise selection (see Fig 8). Consequently SCAD and ALASSO have rather large values of the ‘mean squared error’ mse, which is defined as the average squared difference between the criterion obtained with a specific method and the best criterion. AR performs very well in terms of mse which means that even when it does not give the model with the minimal criterion it still gives a model which is very close to the best.

**Table 2:** Third simulation on linear regression. Comparison of AR with Stepwise (SW), SCAD and multi-step adaptive LASSO (ALASSO) in case of **linear regression** for two scenarios (*p* = 50 and *p* = 500). For the first scenario we consider only BIC, for the second high-dimensional scenario both BIC and mBIC. To assess the quality of classification we report the average over 200 simulation runs of the following quantities: Power, number of false positives (FP), false discovery rate (FDR) and number of misclassifications (Mis). The next two statistics quantify for each procedure the performance in terms of minimizing the criterion: the mean over the squared differences with the criterion of the best method (mse) and the percentage of simulation runs in which a procedure gave the model with the smallest criterion (best). Finally we report the average computational time for one replication (time). For each quantity the best method is printed in bold.

	Power	FP	FDR	Mis	mse	best	time (sec)
*p* = 50, Criterion: BIC							
SW	**0.405**	0.76	0.13	6.70	0.02	**0.97**	1568
SCAD	0.401	0.69	0.12	**6.68**	0.57	0.60	**79**
AR	0.403	0.74	0.13	6.71	**0.01**	0.90	89
ALASSO	0.396	**0.67**	**0.12**	6.71	0.20	0.69	1471
*p* = 500, Criterion: BIC							
SW	0.350	12.30	0.58	28.53	3.48	**0.62**	4429
SCAD	0.334	**7.36**	**0.44**	**24.02**	182.60	0.00	**120**
AR	0.347	10.40	0.54	26.75	**0.90**	0.48	181
ALASSO	**0.352**	9.58	0.50	25.78	26.90	0.01	2530
*p* = 500, Criterion: mBIC							
SW	**0.127**	0.10	0.04	**21.94**	**0.06**	**0.96**	4909
SCAD	0.119	**0.09**	**0.03**	22.12	1.29	0.72	**123**
AR	0.125	0.10	0.04	21.97	0.09	0.86	184
ALASSO	0.125	0.12	0.03	22.00	0.76	0.70	2538

In terms of classification the four methods perform fairly similar, where in particular AR gives results which are extremely close to stepwise selection. The biggest differences can be observed for the high-dimensional scenario with *p* = 500, where as expected BIC tends to select too large models. Interestingly SCAD provides here the smallest number of false positives resulting in the lowest average number of misclassifications. However, this simply means that BIC is not the ideal selection criterion in a sparse high-dimensional setting, and SCAD benefits from not having models along its search path which minimize BIC. Performing selection with mBIC yields a much better control of Type I error rate and here the four methods behave rather similarly in terms of classification.

In summary one might say that for linear regression AR gives results which are similar to stepwise selection, but the procedure is much faster with a runtime which is fairly close to that of SCAD.

### Generalized linear model

In this section we discuss Poisson regression as a particular case of a generalized linear model to illustrate the usefulness of the adaptive ridge procedure beyond linear regression. There is nothing particularly special about Poisson regression, other examples like logistic regression or multinomial regression can be approached quite similarly. As the weighted ridge problem associated with this type of model has no closed-form solution one needs some iterative numerical algorithm for optimization, like gradient descent, Newton-Raphson or Marquardt, but otherwise the AR procedure defined by Eqs (4) and (8) can be directly applied.

The classical Poisson regression problem is of the form $y_{i}∼P\left(\mu_{i}\left(\beta \right)\right)$ where *μ*_*i*_(***β***) = exp(***X***_*i*_ *β*) with $y,\mu \in R^{n}$, $X\in R^{n\times p}$, and $\beta \in R^{p}$. In order to maximize the *L*_0_ penalized log-likelihood of the problem, we introduce for any penalty *λ* ≥ 0 and weight vector $w\in R^{p}$ the following weighted ridge penalized log-likelihood:

$$\begin{array}{c} \ell \left(\beta ;\lambda ,w\right)=\text{const.}+\beta^{T}X^{T}y-u^{T}\mu \left(\beta \right)-\frac{1}{2}\lambda \beta^{T}\text{diag}\left(w\right)\beta \;, \end{array}$$ (13)

where $\text{u}\in R^{n}$ is an all-one column-vector. For given *λ* ≥ 0 we want to maximize this quantity using the Newton-Raphson algorithm. Simple computations give the first two derivatives of ℓ(***β***; *λ*, ***w***),

$$\begin{array}{c} \nabla \ell \left(\beta ;\lambda ,w\right)=X^{T}\left(y-\mu \left(\beta \right)\right)-\lambda \text{diag}\left(w\right)\beta ; \end{array}$$ (14)

$$\begin{array}{c} \text{Hess}\;\ell \left(\beta ;\lambda ,w\right)=-X^{T}\text{diag}\left(\mu \left(\beta \right)\right)X-\lambda \text{diag}\left(w\right). \end{array}$$ (15)

Maximizing Eq (13) can therefore be done iteratively using the following update for ***β***:

$$\begin{array}{c} \beta \leftarrow \beta -\text{Hess}\;\ell {\left(\beta ;\lambda ,w\right)}^{-1}\nabla \ell \left(\beta ;\lambda ,w\right). \end{array}$$ (16)

Accordingly a solver for the weighted Poisson regression model can be implemented in R as follows:

      for (iter in 1:itermax) {

        mu = exp(X\%*\%beta)[,1]

        grad = crossprod(X,y-mu)-pen*w*beta

        hess=-crossprod(X,mu*X)-pen*diag(w)

        beta = beta-solve(hess,grad)

      }

We want to point out that the resulting code for adaptive ridge is compact and extremely easy to understand and to implement. This is in stark contrast to the available LASSO implementation of the same problem [39] which uses a rather delicate coordinate descent algorithm. We believe that this is one of the greatest advantages of the AR procedure that for many different applications it provides a very easy way to perform model selection based on a non-convex penalization scheme.

To evaluate the performance of the AR procedure for Poisson regression we consider again two simulation scenarios with *p* = 50 and *p* = 500, where now count data are simulated from Poisson regression models of size *k* = 10 and *k* = 25, respectively. All other aspects of this simulation study are identical with the last simulation study on linear regression, where we compare again AR with stepwise regression, SCAD and ALASSO. The results are summarized in Table 3, which are qualitatively quite similar to the corresponding results from linear regression. In terms of classification there is no huge difference in performance between the four methods except for selection with BIC in the second scenario. Once again SCAD and ALASSO have on average a smaller number of misclassifications than stepwise search and AR, which reflects the fact that in a sparse high-dimensional setting it is not ideal to perform selection with BIC. Like in case of linear regression stepwise selection tends to perform best with respect to minimizing the selection criterion, closely followed by AR, whereas ALASSO and specifically SCAD tend to give models with substantially larger criteria.

**Table 3:** Simulation on Poisson regression. Comparison of AR with Stepwise (SW), SCAD and multi-step adaptive LASSO (ALASSO) in case of **Poisson regression** for two scenarios (*p* = 50 and *p* = 500). For the first scenario we consider only BIC, for the second high-dimensional scenario both BIC and mBIC. To assess the quality of classification we report the average over 200 simulation runs of the following quantities: Power, number of false positives (FP), false discovery rate (FDR) and number of misclassifications (Mis). The next two statistics quantify for each procedure the performance in terms of minimizing the criterion: the mean over the squared differences with the criterion of the best method (mse) and the percentage of simulation runs in which a procedure gave the model with the smallest criterion (best). Finally we report the average computational time for one replication (time). For each quantity the best method is printed in bold.

	Power	FP	FDR	Mis	mse	best	time (sec)
*p* = 50, Criterion: BIC							
SW	0.431	0.82	0.15	6.51	**0.02**	**0.95**	3367
SCAD	0.420	**0.73**	**0.14**	6.54	0.98	0.57	**135**
AR	0.423	0.79	0.14	6.56	0.03	0.83	2098
ALASSO	**0.428**	0.77	0.14	**6.48**	0.22	0.68	1523
*p* = 500, Criterion: BIC							
SW	0.337	6.84	0.44	23.42	1.82	**0.68**	10184
SCAD	0.328	**5.08**	**0.37**	**21.87**	190.00	0.00	**213**
AR	0.341	6.02	0.41	22.51	**1.54**	0.42	7931
ALASSO	**0.348**	5.58	0.38	21.88	18.70	0.06	2659
*p* = 500, Criterion: mBIC							
SW	**0.216**	0.47	0.07	20.06	**0.94**	**0.89**	10587
SCAD	0.193	0.53	0.08	20.71	93.00	0.25	**212**
AR	0.213	**0.39**	**0.06**	**20.05**	2.54	0.63	7906
ALASSO	0.207	0.43	0.07	20.24	7.41	0.40	2668

The biggest difference to the results from linear regression is concerned with runtime, where AR has now lost its big advantage compared with stepwise regression and ALASSO. On the contrary, AR now actually tends to be slower than ALASSO. However, this comparison is not entirely fair because ALASSO uses the lqa R-package which is based on a highly efficient implementation in C, whereas AR uses only the extremely simple R code sketched above to solve the weighted Poisson regression problem. It is evident that an efficient C implementation of this solver could speed up the procedure substantially, but this is not the main focus of this article. On the contrary, our emphasis is that even such a simple implementation using only a few lines of R-code remains competitive and provides very good results in terms of classification. The biggest advantage is that AR can be easily adapted to more complicated models as we will see in the next section.

### Least squares segmentation

The AR procedure can be applied in contexts which go beyond regression models, like for example for least squares segmentation of a one-dimensional signal which was recently applied in the context of analyzing pathological patterns of DNA in tumor tissues [29]. We want to improve on the original publication by deriving explicit recursive formulas for solving the weighted ridge problem rather than relying on (sparse) LU decompositions. As a result, our approach is much faster than the original one.

Let $y\in R^{n}$ denote *n* measurements which are spatially (or temporally) ordered. Then the problem of segmentation can be formalized by introducing *L*_0_ penalties for changing the estimated mean between neighboring measurements,

$$\begin{array}{c} \hat{\mu }=\arg\min_{\mu \in R^{n}}\{\sum_{i=1}^{n}{\left(y_{i}-\mu_{i}\right)}^{2}+\lambda \sum_{i=1}^{n-1}1\left(\mu_{i}\neq \mu_{i+1}\right)\}\;, \end{array}$$ (17)

where 1(·)∈{0,1} is the indicator function. According to Remark 1 this fits into our context as a slightly generalized version of the penalized contrast Eq (1), and like in [29] we introduce the following weighted ridge square loss as a generalization of Eq (3):

$$\begin{array}{c} \text{SL}\left(\mu ;\lambda ,w\right)=\sum_{i=1}^{n}{\left(y_{i}-\mu_{i}\right)}^{2}+\lambda \sum_{i=1}^{n-1}w_{i}{\left(\mu_{i+1}-\mu_{i}\right)}^{2}\;. \end{array}$$ (18)

For the corresponding AR procedure we again start with the initial weights ***w***^(0)^ ≃ 1 and for *k* ≥ 1 perform the iterations

$$\begin{array}{c} \mu^{\left(k\right)}=\arg\min_{\mu \in R^{n}}\text{SL}(\mu ;\lambda ,w^{\left(k-1\right)})\;,\;w_{i}^{\left(k\right)}={\left(\delta^{2}+{\left(\mu_{i+1}^{\left(k\right)}-\mu_{i}^{\left(k\right)}\right)}^{2}\right)}^{-1}\;. \end{array}$$ (19)

The computations of Eq (19) can be easily solved analytically by considering the derivatives of *SL*(***μ***; *λ*, ***w***). Minimization of the loss function then corresponds to solving the following set of linear equations:

$$\begin{array}{c} \left\{ \begin{array}{c} \left(y_{1}-\mu_{1}\right)+\lambda w_{1}\left(\mu_{2}-\mu_{1}\right)=0\\ \left(y_{2}-\mu_{2}\right)+\lambda w_{2}\left(\mu_{3}-\mu_{2}\right)-\lambda w_{1}\left(\mu_{2}-\mu_{1}\right)=0\\ \vdots \\ \left(y_{n}-\mu_{n}\right)-\lambda w_{n-1}\left(\mu_{n}-\mu_{n-1}\right)=0 \end{array}\right. \end{array}$$ (20)

In [29] it was suggested to solve this problem using an efficient sparse LU decomposition. Here we provide a dramatically faster alternative which allows to recursively compute the solution. For *i* = 1, …, *n* − 1, let us write *μ*_*i*_ = *a*_*i*_ + *b*_*i*_ *μ*_*i*+1_ where $a_{i},b_{i}\in R$. From the linear equations above we obtain

$$\begin{array}{ccccc} a_{1} & = & \frac{y_{1}}{1+\lambda w_{1}} & b_{1}=\frac{\lambda w_{1}}{1+\lambda w_{1}} & i=1;\\ a_{i} & = & \frac{y_{i}+\lambda w_{i-1}a_{i-1}}{D_{i}} & b_{i}=\frac{\lambda w_{i}}{D_{i}} & 1<i<n\;, \end{array}$$ (21)

with *D*_*i*_ = 1 + *λw*_*i*_ + *λw*_*i*−1_(1 − *b*_*i*−1_), and finally

$$\begin{array}{cccc} \mu_{n} & = & \frac{y_{n}+\lambda w_{n-1}a_{n-1}}{1+\lambda w_{n-1}\left(1-b_{n-1}\right)} & i=n\;,\\ \mu_{i} & = & a_{i}+b_{i}\mu_{i+1} & i<n\;. \end{array}$$ (22)

Using these recursive formulas one can hence perform one update step of Eq (19) in O(n). Alternatively, one can use dynamic programming to find the best solution of Eq (17) with at most *k*_max_ ≥ 1 segments in $O(k_{\text{max}}\times n^{2})$. Such a strategy is for example explained in [40] and implemented in the Segmentor3IsBack R package [41].

**Fig 9:**
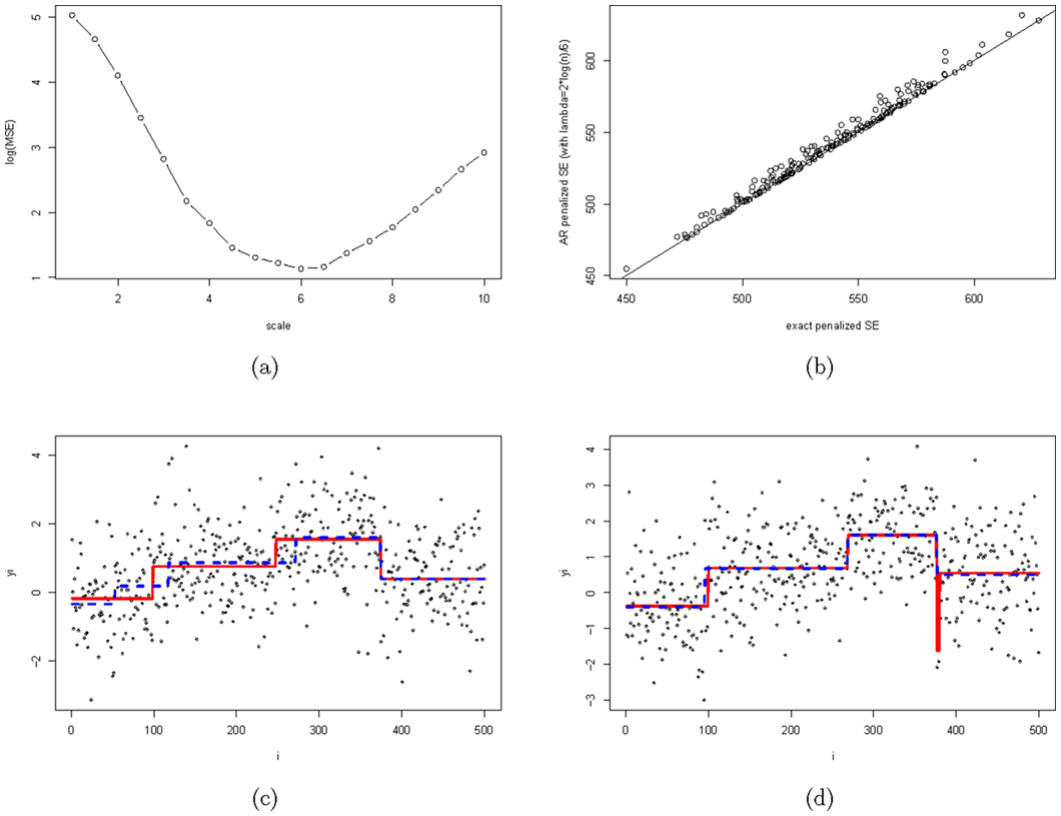
Comparison of exact segmentation (*λ* = 2 log *n*) and adaptive ridge segmentation ($\tilde{\lambda }=\lambda /\text{scale}$). Panel (a) shows the calibration of the rescaling parameter which leads to scale = 6. Panel (b) compares the exact penalized SE to the one obtained through AR with *λ* = 2log(*n*)/6. Panels (c) and (d) illustrate the segmentation output for two specific instances where one observes some disagreement between exact (red solid line) and AR segmentation (blue dashed line).

In order to validate the adaptive ridge approach in the context of least squares segmentation we will compare its performance with the exact approach in a small simulation study. We consider a simple Gaussian design with *n* = 500 consecutive measurements and three breakpoints at positions 100, 250 and 375. Based on a Gaussian model 200 data sets were generated with mean values −0.3, 0.7, 1.5, 0.5 in the four different segments, and a common standard deviation of *σ*^2^ = 1.0. After performing some calibration of the parameter $\tilde{\lambda }$ using the previously discussed warm start method of AR (Fig 9a) we decided upon using the AR penalty $\tilde{\lambda }=\lambda /6$, where *λ* = 2log(*n*) is the penalty of the original criterion. This rescaling factor appeared to be quite stable for various scenarios, though perhaps increasing slightly with growing *n* (data not shown).

We can see in Fig 9b a comparison of the SE penalized criterion obtained both by exact computations and the AR method. AR clearly gives good results, although sometimes suboptimal. In Fig 9c and 9d we give two examples of such suboptimal situations: in Fig 9c, AR has two misplaced breakpoints and the selection of an additional one. In Fig 9d AR missed one very small segment that was considered relevant by the exact approach. Thus although AR did not find the optimal model in terms of the criterion, its solution is in fact closer to the underlying true model. Given the general good performance of AR one might conclude that due to its efficiency it might be preferable to looking for the exact solution particularly for large scale problems.

### Real Data Analysis

To illustrate the applicability of AR in practice we want to reanalyze a GWAS on metabolic traits from a Finnish cohort [36]. Our analysis is based on the data provided by dbGaP (see Acknowledgements) which does not coincide entirely with the data used for the original publication. On the one hand dbGaP provides data for a slightly larger number of patients (e.g. *n* = 4843 for LDL) compared with the data set used in [36] (*n* ≤ 4518 for LDL). On the other hand the covariates of smoking status and alcohol intake, which play a crucial role for some of the metabolic traits, are not provided by dbGaP. One thus has to be a little bit careful when comparing our results with those of [36], although in general there is quite good agreement.

In the original analysis LDL was the trait for which the greatest number of associated SNPs was reported. Consequently this is the trait which is most interesting for us, because the more complex a trait the more gain can be expected from applying a model selection approach [9, 42]. Furthermore according to [36] the influence of smoking and alcohol intake on LDL was not too large, so it is not a huge problem that we are lacking that information. For these reasons we will focus in our presentation on the trait LDL.

Sabatti et al. [36] performed data analysis by applying main effect models for each marker separately while correcting for sex, pregnancy status, oral contraceptive use and BMI. The problem of potential population stratification was addressed by using a genomic-control parameter. We are using in our analysis linear regression models with the same four patient related covariates (sex, pregnancy status, oral contraceptive use and BMI) and add the first four principal components of the genotype matrix to correct for population stratification. The resulting 8 covariates will be not under selection in our AR approach. Sabatti et al. applied single marker tests for each SNP using only data from those individuals where genotyping was successful. Therefore the number of individuals used for analysis is for some SNPs much smaller than *n* = 4518 (with a minimum of *n* = 4268 for the reported associations). For the AR approach one needs complete genotype data for all SNPs. To achieve this we performed imputation of missing values using Beagle 4.1 [43].

Concerning multiple testing correction Sabatti et al. applied the Benjamini Hochberg procedure at a nominal level of 0.05 and then argued that equivalently they might use a significance threshold of 5 × 10^−7^ which corresponds approximately to a Bonferroni correction at nominal level 0.16. The mBIC criterion we are going to adopt is of the form

$$\begin{array}{c} \text{mBIC}\left(c\right)=-2\log L\left(M\right)+|M|\log(np^{2}c^{-2}) \end{array}$$

where *c* can be interpreted from a Bayesian point of view as the a priori expected number of causal SNPs. From a frequentist perspective the mBIC criterion is closely connected with the Bonferroni multiple testing rule, where in [7] one can find an approximate formula of the family wise error rate (FWER) to be expected when applying mBIC(*c*) for *m* independent regressors and *n* individuals. In our case the typically recommended choice of *c* = 4 yields FWER = 0.008, which appears to be extremely conservative compared with the multiple testing correction used by Sabatti et al. Using mBIC with *c* = 10 gives a FWER of 0.02 which is still rather conservative, whereas *c* = 23 yields FWER = 0.05.

Table 4 reports all SNPs detected by AR when trying to minimize mBIC(10). Before applying AR we preselected SNPs with marginal p-value smaller than 0.001 and we did not include SNPs from the X chromosome. Given the uncertainty of the relationship $\lambda =4\tilde{\lambda }$ from Theorem 1 in case of correlated regressors we performed AR with a number of scaling parameters ranging from 2 to 6. Among all different models thus obtained we report the one which minimizes mBIC(10). From the 8 SNPs we have detected with this procedure 5 coincide with SNPs reported in [36] and 2 are in very close proximity to the SNP *rs693* also reported in [36]. Thus our detections cover all autosomal SNPs from Sabatti et al. and include one additional SNP *rs207150* lying on chromosome 1. Note that this SNP has a marginal p-value of 5.6E-07 and would therefore not have been selected applying the threshold 5 × 10^−7^ suggested by Sabatti et al. This goes along with the fact that model based analysis will typically have larger power to detect SNPs than single marker tests, even when the selection criterion guarantees that the type I error rate is controlled at a stricter level (see [9] for a comprehensive discussion of this topic). Applying AR to minimize mBIC(23) gave the same result as mBIC(10), whereas the extremely conservative choice of mBIC(4) resulted only in the selection of 2 SNPs (see Table 4).

**Table 4:** Real Data Analysis. Results from GWAS analysis for the trait LDL. The first three columns provide the identifier, chromosome and position of each SNP detected by AR with a penalty mBIC(10). Here 10 is the a priori expected number of causal SNPs whereas the standard mBIC uses *c* = 4. The last three columns show the p-values of marginal tests including covariates as specified in the text, the two SNPs selected by standard mBIC and finally information about the SNPs reported by Sabatti et al. [36]. The symbol • refers to exactly the same SNP, * marks two SNPs which are in close proximity to *rs693* reported in [36], and # is one representative of a region on chromosome 11 for which [36] reports 5 significant SNPs.

SNP	Chr	Position	p-value	mBIC(4)	Sabatti
*rs207150*	1	55579053	5.6E-07		
*rs646776*	1	109620053	1.1E-14	•	•
*rs4844614*	1	205941798	1.0E-07		•
*rs6728178*	2	21047434	2.9E-09		*
*rs1713222*	2	21124828	5.7E-07		*
*rs174556*	11	61337211	3.4E-07		• #
*rs11668477*	19	11056030	3.7E-08	•	•
*rs157580*	19	50087106	2.1E-07		•

## Discussion

In this paper we have introduced the adaptive ridge procedure AR, an iterative procedure whose purpose is to solve *L*_0_ penalized problems via weighted ridge optimization. The approach, recently suggested by [29] in the particular context of least squares segmentation, is very similar to the iterative adaptive LASSO procedure introduced in [23, 24], with the noticeable difference that AR requires at each iteration to solve a weighted ridge problem instead of the weighted LASSO. As a result, the practical implementation of the adaptive ridge is often dramatically simpler than its adaptive LASSO counterparts.

The possibility of a multi-step adaptive LASSO which has been widely discussed at a conceptual level [17, 23, 24]. However, up to our knowledge there does not exist any software package which has implemented multi-step adaptive LASSO in the sense that steps are repeated till convergence of weights has been achieved. In our own attempts to implement multi-step adaptive LASSO based on existing software for adaptive LASSO we observed a number of problems (like programs crashing or numerical instabilities), so developing ALASSO for different applications appears to be not a completely trivial task. On the other hand adaptive ridge turned out to be running stable for all models we looked at. In principle adaptive ridge should also be computationally faster than the ALASSO, but we only see this in our simulations on linear regression. For Poisson regression we do not have an optimized implementation of the weighted regression problem to be solved in each step of the iteration which explains why we AR is here somewhat slower than the five-step adaptive LASSO procedure based on the lqa R-package.

It was pointed out in [29] that the adaptive ridge approach clearly performs very well in practice, though any theoretical justifications of that behavior was missing. In this paper we partially addressed this problem by studying the dynamics of AR in the particular case of orthogonal linear regression (with known variance). In this context we derived explicit conditions for the convergence of AR and proved that the adaptive ridge penalty needs to be four times smaller than the original *L*_0_ penalty to give the same results. According to our simulations this scaling factor of 1/4 worked quite well also in case of non-orthogonal linear regression, as long as the correlation between covariates was not too high. In case of highly correlated regressors, as well as for *p* ≫ *n*, further investigation might be necessary, but in general such rescaling offers a natural way to select adaptive ridge penalties by targeting classical *L*_0_ penalty schemes like AIC and BIC, or in a high-dimensional setting the more recently suggested mBIC.

In principle adaptive ridge should be also computationally faster than the multi-step adaptive LASSO, although we do not quite see this advantage in our presentation because on the one hand we do not have an optimized implementation Furthermore the AR procedure, just like the LASSO, allows to take advantage of warm starts to compute efficiently the entire solution surface for a sequence of penalties. This gives the possibility to select the most appropriate penalty of AR without any need to know the rescaling scheme. Note that for the adaptive ridge we have to consider increasing penalty values, whereas for the LASSO one usually considers decreasing penalty values.

In summary the AR procedure suggested in this paper is quite straightforward to understand and implement, can be easily combined with iterative optimization procedures like Newton-Raphson, and offers efficient ways to compute entire solution surfaces. We hope that this paper could be a first step to learn more about the theoretical properties of this method, which definitely seems to be worth of further investigation.

## Supporting Information

S1 Proof Proof of Theorem 1. (PDF)
