# Supplementary material for: An Adaptive Ridge Procedure for L0 Regularization
Source: PLoS One. 2016 Feb 5;11(2):e0148620. doi: 10.1371/journal.pone.0148620 (PMC4743917; doi:10.1371/journal.pone.0148620)
Supplement: S1 Proof — (PDF) [file pone.0148620.s001.pdf]

Web Supplement for:
An adaptive ridge procedure for L_0 regularization

Florian Frommlet[1,*], Grégory Nuel[2]
1 Department of Medical Statistics (CEMSIIS), Medical University of Vienna, Spitalgasse 23, A-1090 Vienna, Austria
2 National Institute for Mathematical Sciences (INSMI), CNRS, Stochastics and Biology Group (PSB), LPMA UMR CNRS 7599, Université Pierre et Marie Curie, 4 place Jussieu, 75005 Paris, France
∗ E-mail: Florian.Frommlet@meduniwien.ac.at

Shrinkage properties for the orthogonal case

We will give here the proof of Theorem 1, that under orthogonality performing AR with $\tilde{\lambda}$ is equivalent to minimizing the original penalized likelihood criterion with $\lambda = 4\tilde{\lambda}$.

Assume that $p \leq n$ and that the design matrix fulfills $\boldsymbol{X}^T\boldsymbol{X} = n\boldsymbol{I}_p$, where $\boldsymbol{I}_p$ is the identity matrix of dimension p. Then the usual maximum likelihood estimate of β for the saturated model becomes $\hat{\boldsymbol{\beta}} = \frac{1}{n}\boldsymbol{X}^T\boldsymbol{y}$, and the original penalized likelihood criterion evaluated at the maximum likelihood estimate of any given model can be rewritten as

$$C_{\lambda,0}\left(\hat{\boldsymbol{\beta}}^M\right) = \frac{1}{\sigma^2}\left(\boldsymbol{y}^T\boldsymbol{y} - n\sum_{j\in M}\hat{\beta}_j^2\right) + \lambda|M| \ . \tag{1}$$

Thus the penalized likelihood is minimized when all those regressors enter the model for which

$$\hat{\beta}_j^2 > \lambda\sigma^2/n \ , \tag{2}$$

which results in the well known fact that under orthogonality the model selection procedure defined by (1) is nothing else but a thresholding procedure for the individual coefficients. Note that the whole argument relies upon the fact that in case of orthogonality the coefficients $\hat{\beta}_j$ are estimated independently from each other.

We next argue that AR also becomes a simple thresholding procedure under orthogonality. First note that the dynamic system (11) of the manuscript can be rewritten as

$$\tilde{\beta}_j^{(1)} = \frac{1}{1+K}\hat{\beta}_j \ , \quad \tilde{\beta}_j^{(k)} = \frac{1}{1+\frac{K}{\delta^2+(\tilde{\beta}_j^{(k-1)})^2}}\hat{\beta}_j \ , \quad j = 2,\dots,p \tag{3}$$

where we define $K \triangleq \tilde{\lambda}\sigma^2/n$. Thus we have for each coefficient a one-dimensional dynamic system independent of the other coordinates, which is easy to solve. Equation (3) already indicates the shrinkage of the limit $\tilde{\beta}_j$ compared with the ML estimate $\hat{\beta}_j$. The stationary points of the sequence $\tilde{\beta}_j^{(k)}$ can be found by solving the equation

$$\tilde{\beta}_j\left(1 + \frac{K}{\delta^2+\tilde{\beta}_j^2}\right) = \hat{\beta}_j \ . \tag{4}$$

For the sake of notational convenience let's write $x_k = \tilde{\beta}_j^{(k)}$. We thus study the dynamic system

$$x_k = \frac{\hat{\beta}_j}{f(x_{k-1})}, \quad \text{with} \ \ f(x) = 1 + K(\delta^2+x^2)^{-1} \ , \tag{5}$$

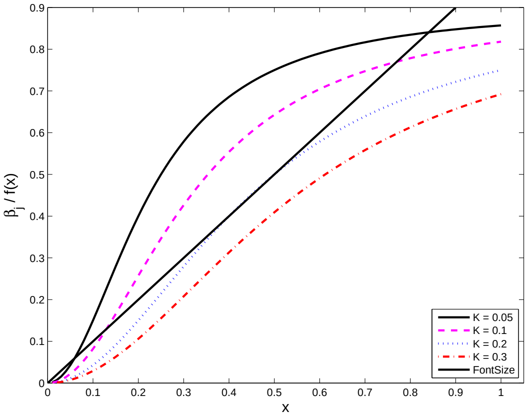

Figure 1. Function which determined the dynamic system (5) for $\delta = 10^{-5}$ and $\hat{\beta}_j = 0.9$.

which is illustrated in Figure 1. As long as $K > 8\delta^2$ (which is essentially always the case) the function $xf(x)$ has two positive local extrema. The dynamic system (5) has only one stationary point x_I when the function value of its positive local minimum x_* is larger than $\hat{\beta}_j$, that is

$$x_* f(x_*) > \hat{\beta}_j, \text{ with } x_*^2 = \frac{K}{2} - \delta^2 + \frac{1}{2}\sqrt{(K - 2\delta^2)^2 - 4\delta^2}\,. \tag{6}$$

If $\delta \ll K$ this roughly means that $\hat{\beta}_j < 2\sqrt{K}$. In that case it is easy to see that the only stationary point x_I is attractive, and one has $x_k \to x_I \approx 0$ (see Figure 1 for $K = 0.3$).

The other common situation occurs when the inequality in (6) changes, that is when essentially $\hat{\beta}_j > 2\sqrt{K}$. Then it holds that (4) has three solutions $x_I < x_{II} < x_{III}$. Standard arguments from the theory of dynamical systems show that x_I and x_{III} are attractive, that is for $x_1 < x_{II}$ one has $x_k \to x_I$, otherwise if $x_1 > x_{II}$ then $x_k \to x_{III}$ (see Figure 1 for $K = 0.1$ and $K = 0.05$). Note that x_I, x_{II} and x_{III} are the roots of a polynomial of third degree for which explicit formulas are available.

In the exceptional case where there are only two stationary points the dynamic is such that for $x_1 < x_{II}$ one has again $x_k \to x_I$, but for $x_1 > x_{II}$ one has $x_k \to x_{II}$. Thus x_{II} is a saddle point (see Figure 1 for $K = 0.2$).

Convergence of $x_k \to x_I$ can be interpreted as $\tilde{\beta}_j = 0$, although $0 < x_I \approx \delta^2 \hat{\beta}_j / K$. However, numerically this is small enough to be indistinguishable from zero as long as δ is sufficiently small. Thus from a model selection perspective convergence towards x_I indicates that a coefficient has been excluded, whereas the limit x_{III} corresponds to regressors which have been included in the model. Furthermore equation (4) shows the amount of shrinkage that a regression coefficient suffers from AR. The larger x_{III} and the smaller K, the less shrinkage.

Two conditions have to be fulfilled that a regressor is selected by AR. Firstly the dynamical system of the component must have three fixed points, which corresponds to the condition that $K < \hat{\beta}_j^2/4$. Secondly it is then necessary that $x_1 > x_{II}$. Remember that AR computes in its first step $\tilde{\boldsymbol{\beta}}^{(1)}$ by standard ridge regression, and therefore $x_1 = \hat{\beta}_j/(1 + K)$. On the other hand a very good approximation of x_{II} can be

obtained by letting $\delta = 0$ in (5) and then solving the corresponding stationary equation, which results in

$$x_{II} \approx \hat{\beta}_j/2 - \sqrt{\hat{\beta}_j^2/4 - K}\ .$$

As long as $K < 1$ it then always holds that $x_1 > x_{II}$, and it follows that the dynamic of AR under orthogonality is completely determined by the number of fixed points for each regressor. To summarize, under orthogonality AR becomes a thresholding procedure where a regressor is selected in case of

$$\hat{\beta}_j^2 > 8\lambda\sigma^2/n\ . \tag{7}$$

Comparing conditions (2) and (7) then yields Theorem 1. The condition $K < 1$ is per definition of K equivalent to $\tilde{\lambda} < n/\sigma^2$.
